# Menopausal Status and Physical Activity Are Independently Associated With Cardiovascular Risk Factors of Healthy Middle-Aged Women: Cross-Sectional and Longitudinal Evidence

**DOI:** 10.3389/fendo.2019.00589

**Published:** 2019-08-30

**Authors:** Sira Karvinen, Matthew J. Jergenson, Matti Hyvärinen, Pauliina Aukee, Tuija Tammelin, Sarianna Sipilä, Vuokko Kovanen, Urho M. Kujala, Eija K. Laakkonen

**Affiliations:** ^1^Gerontology Research Center, Faculty of Sport and Health Sciences, University of Jyväskylä, Jyväskylä, Finland; ^2^Department of Rehabilitation Medicine, Medical School, University of Minnesota, Minneapolis, MN, United States; ^3^Pelvic Floor Research and Therapy Unit, Department of Obstetrics and Gynecology, Central Finland Central Hospital, Jyväskylä, Finland; ^4^LIKES Research Centre for Physical Activity and Health, Jyväskylä, Finland; ^5^Faculty of Sport and Health Sciences, University of Jyväskylä, Jyväskylä, Finland

**Keywords:** menopause, cardiovascular disease, physical activity, cholesterol, HDL, LDL, triglycerides, fasting blood glucose

## Abstract

Cardiovascular disease (CVD) is the primary cause of mortality in women in developed countries. CVD risk rises with age, yet for women there is a rapid increase in CVD risk that occurs after the onset of menopause. This observation suggests the presence of factors in the middle-aged women that accelerate the progression of CVD independent of chronological aging. Leisure time physical activity (LTPA) is a well-established protective factor against CVD. However, its role in attenuating atherogenic lipid profile changes and CVD risk in post-menopausal women has not been well-established. The present study is part of the Estrogenic Regulation of Muscle Apoptosis (ERMA) study, a population-based cohort study in which middle-aged Caucasian women (47–55) were classified into pre-menopausal, peri-menopausal, and post-menopausal groups based on follicle stimulating hormone levels and bleeding patterns. Comprehensive questionnaires, laboratory visits, anthropometric measurements, and physical activity monitoring by accelerometers were used to characterize the menopausal groups and serum lipid profiles were analyzed to quantify CV (cardiovascular) risk factors. Based on our findings, LTPA may attenuate menopause-associated atherogenic changes in the serum CV risk factors of healthy middle-aged women. However, LTPA does not seem to entirely offset the lipid profile changes associated with the menopausal transition.

## Introduction

Cardiovascular disease (CVD) is the primary cause of mortality in women in developed countries (1). It has long been noted that CVD seems to be rare among women younger than 45 years of age, but by age 70, women experience CVD at the same rate as their male counterparts (2). Furthermore, studies in pre-menopausal women show that hysterectomy is associated with higherrisk of CVD and stroke (3–5) and increments in blood lipids and lipoprotein cholesterol levels (6–8). In addition, menopause associates with unfavorable changes in body composition, leading to increased fat tissue mass, and decreased lean body mass (9) as well as unfavorable changes in the blood lipid profile [i.e., increased serum total cholesterol, low-density lipoprotein cholesterol (LDL-C) and triglyceride levels and decreased high-density lipoprotein cholesterol (HDL-C) levels] (2). Therefore, menopause has been seen as one risk factor for developing metabolic syndrome and CVD, which may occur even independent of chronological aging (10–12).

Yet, some studies have reported an inconsistent association of menopause with the serum cardiovascular (CV) risk factors, including total cholesterol, LDL-C, HDL-C, triglycerides, and fasting blood glucose. To our knowledge at least nine cross-sectional (7, 13–20) and four longitudinal (21–24) studies are currently available with measurements of all or most of the above-mentioned clinically measured CV risk factors. Total cholesterol and LDL-C were found to be higher in post-menopausal women compared to pre-menopausal women by all the listed cross-sectional studies except one (16) in which difference in the LDL-C level was not significant. The results were much more variable for HDL-C, which two studies found to be lower (13, 17) and two higher (7, 20) in post-menopausal women compared to pre-menopausal, while five studies (14–16, 18, 19) reported no significant difference between menopausal groups. Of the discussed cross-sectional studies, one did not measure triglycerides (16) and five did not find significant group differences (7, 15, 18–20), while three (13, 14, 17) found higher levels for post-menopausal compared to pre-menopausal women. Fasting blood glucose was measured only in three of the studies (15, 17, 20), of which only Cho et al. reported significantly higher levels in post-menopausal women, while others did not find significant group differences. The longitudinal studies over the menopausal transition have also provided partially contradicting results. Jensen et al., Do et al., and Matthews et al. reported upregulation of total cholesterol, LDL-C and triglycerides over the menopausal transition while Abdulnour et al. did not find significant change for any of them. HDL-C was reported to be downregulated by Jensen et al. and Do et al. and upregulated by Matthews et al. and Abdulnour et al. Fasting glucose was measured only in studies by Matthews et al. and Abdulnour et al., which both found it to be upregulated over the menopausal transition. However, studies by Do et al. and Matthews et al. made specific remarks that not all of the results were associated specifically with the menopausal transition, but more likely with aging *per se*. Therefore, it is still controversial whether menopausal status or chronological aging has a more prominent association with serum CV risk factors.

The variability in the results obtained by the previous studies may partially be due to differences in the mean ages of the menopausal groups and in the determination of menopausal status, which in most cases relies on self-reports regarding menstrual cycle. Only six (13, 18, 20, 21, 23, 24) out of the 13 studies used additional hormone measurements to determine menopausal status. Furthermore, there may be confounding factors which have not been taken similarly into account in all studies. For example, only seven of the studies (13, 17, 18, 20, 22–24) attempted to measure potential differences in the physical activity (PA) levels of the study participants, and, of the seven, only Abdulnour et al. (24) used accelerometer first to measure physical activity and then to control differences in PA in their analysis. Therefore, large scale studies with precise determination of the menopausal status and PA are warranted to overcome the variability of the results and to eventually conclude if the menopausal transition has an effect on CV risk factors independent of aging.

The present study is a part of large-scale population-based cohort study, Estrogenic Regulation of Muscle Apoptosis (ERMA) (25), that comprised of healthy women aged 47–55 years and included both cross-sectional (*base-ERMA*) and longitudinal (*core-ERMA*) study designs. The current study had three major objectives. First, it sought to determine whether there are significant differences in serum CV risk factors between groups of middle-aged women classified as pre-menopausal, peri-menopausal, and post-menopausal according to follicle stimulating hormone (FSH) levels and bleeding patterns. Second, it examined the changes in serum CV risk factors over the menopausal transition using a longitudinal study design. Third, it evaluated whether any of the serum CV risk factor differences between study participants could be independently explained by menopausal status and leisure time physical activity (LTPA).

## Results

**Figure 1:**
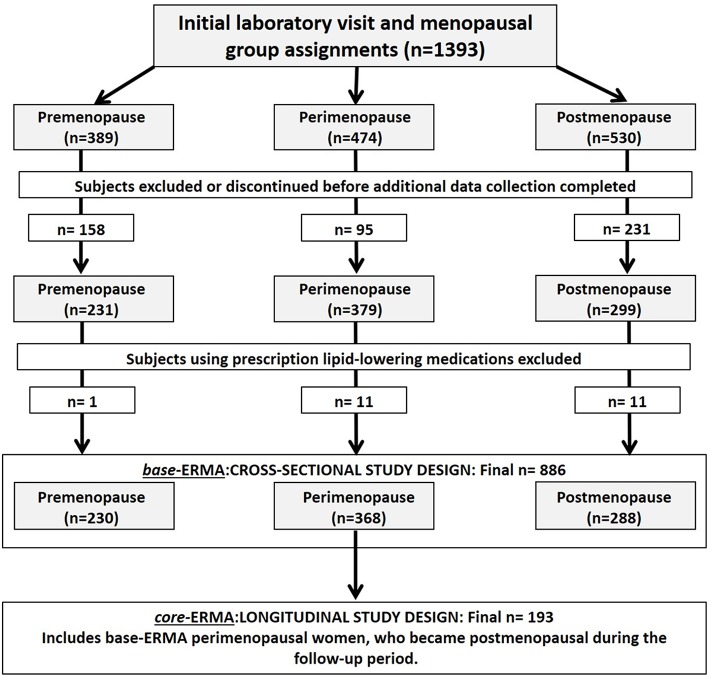
Flow chart of the final subject group determination for the cross-sectional (*base-ERMA*) and longitudinal (*core-ERMA*) study designs.

The current study is part of the ERMA study described previously (25). In brief, the ERMA study is a population-based cohort study comprising of women aged 47–55 years living in the city of Jyväskylä and neighboring municipalities, in Finland. The data presented here includes both cross-sectional (*base-ERMA, n* = 886) and longitudinal (*core-ERMA, n* = 193) study designs (Figure 1). Initially 1,393 participants completed the menopausal group assignments and health screen questionnaire, but between the first lab visit and the following physiological and psychological assessments, 507 participants were excluded or discontinued the study. Furthermore, participants taking prescription lipid-lowering medications (23 participants) were excluded from the final analysis. Finally, 886 women remained in the cross-sectional *base-ERMA* study setup and were used to explore the associations of menopausal status and LTPA with CV risk factors in a population-based, large-scale study design. The peri-menopausal women of the *base-ERMA* study entered into the longitudinal *core-ERMA* study. Of them, 193 went through the natural menopausal transition during the 16 ± 8 month follow-up period. The progression of the menopausal transition was evaluated based on menstrual bleeding diaries and hormone assessments taken in 3–6 month intervals. Therefore, the *core-ERMA* design enabled close follow-up of the menopausal transition from peri-menopause to early post-menopause, minimizing confounding factors.

### *Base-ERMA*: Socio-Demographic and Life-Style Characteristics of the Participants

Socio-demographic and life-style factors including LTPA for each *base-ERMA* group are shown in Table 1. As expected, the mean age in the peri-menopausal group was significantly higher than in the pre-menopausal group (*p* < 0.001), while the post-menopausal participants were significantly older than those in both the pre- and peri-menopausal groups (*p* < 0.001). The highest level of education achieved differed between the groups (*p* = 0.019), with pre- and peri-menopausal women appearing to achieve tertiary (master's or doctoral) levels of education at higher rates, while post-menopausal women were more likely to have achieved a secondary education level. The pre-menopausal group had higher total and lean body mass than the post-menopausal group (*p* < 0.050). Meanwhile, the post-menopausal group had significantly lower body mass index (BMI) and lean body mass than the peri-menopausal group (*p* < 0.050) but did not differ in percent body fat. The post-menopausal women had significantly higher leisure time vigorous PA in accelerometer-based activity measures compared with pre- and peri-menopausal women (*p* < 0.050), yet the groups did not differ in their PA total counts, self-reported LTPA or cardiorespiratory fitness assessed by 6-min walking test.

**Table 1:** Characteristics of the study participants of the cross-sectional study design (*base-ERMA*).

	**Pre-menopause** ***n* = 230**	**Peri-menopause** ***n* = 368**	**Post-menopause** ***n* = 288**	***p*-value**
**Age** [years]	50.64 (1.62)	51.19 (1.91)^‡^	52.54 (1.91)^‡^^¶^	**<0.001**
**Age Range** [years]	47-54	47-55	48-55	
**Education** [*n* (%)]			^†^^;*^	**0.019**
Primary	1 (0.4)	8 (2.2)	7 (2.4)	
Secondary	126 (54.8)	198 (53.8)	181 (62.8)	
Tertiary	103 (44.8)	162 (44.0)	100 (34.7)	
**Cigarette Smoking**				0.837
Never Smoked	153 (66.5)	247 (67.1)	198 (68.8)	
Quit smoking	63 (27.4)	90 (24.5)	71 (24.7)	
Current Smoker	14 (6.1)	31 (8.4)	19 (6.6)	
**Alcoholic drinks/wk**	3.45 (3.17)	4.02 (3.67)	4.02 (4.18)	0.283
**Percent Body Fat** [%]	29.58 (7.12)	31.02 (7.77)	30.46 (7.32)	0.068
**Body mass [kg]**^¤^	70.05 (10.13)	70.37 (11.08)	68.41 (10.99)^^†^^;*^^	**0.050**
**Lean Body Mass** [kg]	45.99 (4.49)	45.23 (5.18)^†^;	44.25 (4.66)^‡*^	**<0.001**
**Body Mass Index** [kg/m^2^]	25.37 (3.29)	25.75 (3.85)	25.01 (3.74)^*^	**0.029**
Normal (<24.9)	113 (49.1)	180 (49.0)	162 (56.3)	
Overweight (25.0-29.9)	95 (41.3)	127 (34.6)	91 (31.6)	
Obese (>30.0)	22 (9.6)	60 (16.3)	35 (12.2)	
**Self-reported leisure time PA** [MET hours/day]	3.77 (3.94)	3.68 (3.80)	3.72 (3.42)	0.825
**Accelerometer measured leisure time PA^§^**				
Sedentary [min/day]	364.58 (45.89)	367.14 (52.44)	361.90 (50.66)	0.394
Light [min/day]	197.37 (40.83)	195.09 (43.59)	198.18 (43.44)	0.681
Moderate [min/day]	34.03 (16.52)	33.91 (17.96)	34.86 (17.79)	0.766
Vigorous [min/day]	4.03 (7.40)	3.86 (6.55)	5.05 (7.82)^†^;^*^	**0.036**
Total counts	4.3 × 10^5^ (1.2 × 10^5^)	4.3 × 10^5^ (1.4 × 10^5^)	4.4 × 10^5^ (1.3 × 10^5^)	0.171
**6-min walking distance** [m]^£^	674.76 (58.71)	666.44 (64.32)	669.60 (58.17)	0.281

*Values are mean (standard deviation) for continuous variables and n (percentage) for categorical variables*.

† p < 0.05 and p <0.001 compared with pre-menopausal women. p < 0.05 and

¶ *p < 0.001 compared with peri-menopausal women*.

§ Missing values = 126,

£ missing values = 76, and

¤ *missing values = 2. Statistically significant findings (p < 0.05) are marked with bold in all of the tables*.

### *Base-ERMA*: Gynecologic and Other Medical Factors

Data related to gynecologic and health factors as well as diseases and medication for each *base-ERMA* group are shown in Table 2. The pre-menopausal group had significantly lower FSH and higher estradiol levels than either the peri- or post-menopausal groups, and the post-menopausal FSH concentration was higher and estradiol level lower than the peri-menopausal group (*p* < 0.001). The groups differed significantly in the use of hormonal forms of contraceptives, with the pre-menopausal group appearing to use at higher rates than the other groups (*p* < 0.001). The groups did not differ in disease incidence or medications used. Fasting blood glucose concentrations were lower in the post-menopausal group than in both the pre-menopausal and peri-menopausal groups (*p* < 0.05).

**Table 2:** Gynecologic and health factors of the study participants of the cross-sectional study design (*base-ERMA*).

	**Pre-menopause** ***n* = 230**	**Peri-menopause** ***n* = 368**	**Post-menopause** ***n* = 288**	***p*-value**
**Follicle stimulating hormone [IU/L]**	7.91 (3.51)	31.81 (21.02)^‡^	83.00 (29.84)^‡^^¶^	**<0.001**
**Estradiol [nmol/L]**	0.621 (0.659)	0.329 (0.262)^‡^	0.142 (0.964^‡^^¶^	**<0.001**
**Hysterectomy**	22 (9.6)	19 (5.2)	22 (7.6)	0.333
**Contraceptive use** [*n* (%)]				**<0.001**
No use	88 (38.3)	198 (53.8)	158 (54.9)	
Former	37 (16.1)	58 (15.8)	56 (19.4)	
Current	105 (45.7)	112 (30.4)	74 (25.7)	
**LDL:HDL ratio**	1.90 (0.77)	1.91 (0.77)	2.00 (1.01)	0.668
**Fasting blood glucose** [mmol/L]	5.20 (0.47)	5.23 (0.58)	5.13 (0.64)^†^^*^	**0.016**
**Systolic BP** [mm Hg]	130.42 (16.85)	131.45 (18.66)	130.66 (16.77)	0.900
**Diastolic BP** [mm Hg]	83.31 (9.48)	83.95 (10.12)	83.55 (9.69)	0.585
**Leptin** [ng/ml]^§^	16.40 (12.67)	16.24 (10.48)	15.88 (11.66)	0.565
***Disease*** [*n* (%)]				
CVD	3 (1.3)	1 (0.3)	1 (0.3)	0.218
Hypertension	25 (10.9)	55 (14.9)	36 (12.5)	0.334
Type 2 diabetes	2 (0.9)	4 (1.1)	2 (0.7)	0.869
***Medications*** [*n* (%)]				
RAAS-acting agents	17 (7.4)	46 (12.5)	27 (9.4)	0.115
β-blockers	16 (7.0)	24 (6.5)	23 (8.0)	0.765
Calcium-channel blockers	5 (2.2)	9 (2.4)	8 (2.8)	0.907
Drugs used in diabetes	0 (0.0)	3 (0.8)	2 (0.7)	0.406

*Values are mean (standard deviation) for continuous variables and n (percentage) for categorical variables*.

† p < 0.05 and

‡ *p < 0.001 compared with pre-menopausal women*.

* p < 0.05 and

¶ *p <0.001 compared with peri-menopausal women*.

§ *Missing values = 7. Statistically significant findings (p < 0.05) are marked with bold in all of the tables*.

### *Core-ERMA*: Characteristics of the Participants

The participant group in the *core-ERMA* study design was comprised of a subset of subjects from the *base-ERMA* design's peri-menopausal group (Figure 1). Of the 368 women, 193 went through the menopausal transition from peri-menopause to early post-menopause in the course of the study thus enabling longitudinal data (Table 3). The characteristics from the *core-ERMA* study closely followed the differences identified in the *base-ERMA* (peri-menopausal vs. post-menopausal group). The FSH level increased and estradiol level decreased after menopause compared to the peri-menopausal values (*p* < 0.010). Body mass and body fat percentage increased and lean body mass decreased during the menopausal transition (*p* < 0.010). The prevalence of CVD or type 2 diabetes did not increase, yet hypertension became more common accompanied by an increase in the use of RAAS-acting agents (*p* < 0.050). Nevertheless, the group mean values of both systolic and diastolic blood pressure slightly decreased over the menopausal transition (*p* = 0.035 and *p* < 0.001, respectively). Of the 193 participants, 12 began to use HT during the follow-up period, yet this did not significantly affect the results (*data not shown*). There was no change in self-reported or accelerometer-measured LTPA.

**Table 3:** Characteristics of the participants of the longitudinal study design (*core-ERMA, n* = 193).

	**Peri-menopause (baseline)**	**Early post-menopause (follow-up)**	***p*-value**
**Follicle stimulating hormone** [IU/L]	36.82 (21.10)	66.49 (34.73)	**<0.001**
**Estradiol [nmol/L]**	0.334 (0.245)	0.263 (0.265)	**<0.001**
**LDL:HDL ratio**	1.84 (0.76)	1.81 (0.73)	0.263
**Fasting blood glucose** [mmol/L]	5.20 (0.54)	5.17 (0.60)	0.569
**Systolic BP [mm Hg]**^§^	131.59 (19.56)	129.71 (18.55)	**0.035**
**Diastolic BP [mm Hg]**^§^	83.62 (10.31)	81.49 (10.05)	**<0.001**
**Percent Body Fat** [%]	30.79 (8.27)	31.84 (7.89)	**<0.001**
**Body mass [kg]**^†^	69.66 (11.23)	70.36 (11.48)	**0.001**
**Lean Body Mass** [kg]	44.91 (5.37)	44.57 (5.23)	**0.002**
**Disease** [*n* (%)]^§^			
CVD	0 (0.00)	0 (0.00)	1.00
Hypertension	25 (13.20)	28 (14.80)	**<0.001**
Type 2 diabetes	2 (1.10)	2 (1.10)	1.00
**Medications** [*n* (%)]			
RAAS-acting agents	19 (9.80)	24 (12.40)	**0.025**
β-blockers	16 (8.30)	19 (9.80)	0.083
Calcium-channel blockers	4 (2.10)	6 (3.10)	0.157
HT-usage (*n*)	0 (0.0)	12 (6.20)	0.317
**Self-reported leisure time PA** [MET hours/day]^¤^	3.66 (3.84)	3.43 (3.77)	0.151
**Accelerometer measured leisure time PA** (total counts)^#^	4.18 × 10^5^ (1.28 × 10^5^)	4.11 × 10^5^ (1.27 × 10^5^)	0.459

Values are mean (standard deviation) for continuous variables and n (percentage) for categorical variables.

§ Missing values = 2,

† missing values = 3,

¤ missing values = 4,

# *missing values = 44. Statistically significant findings (p < 0.05) are marked with bold in all of the tables*.

### *Base*- and *core-ERMA*: Association of Menopausal Status With Serum CV Risk Factors

**Figure 2:**
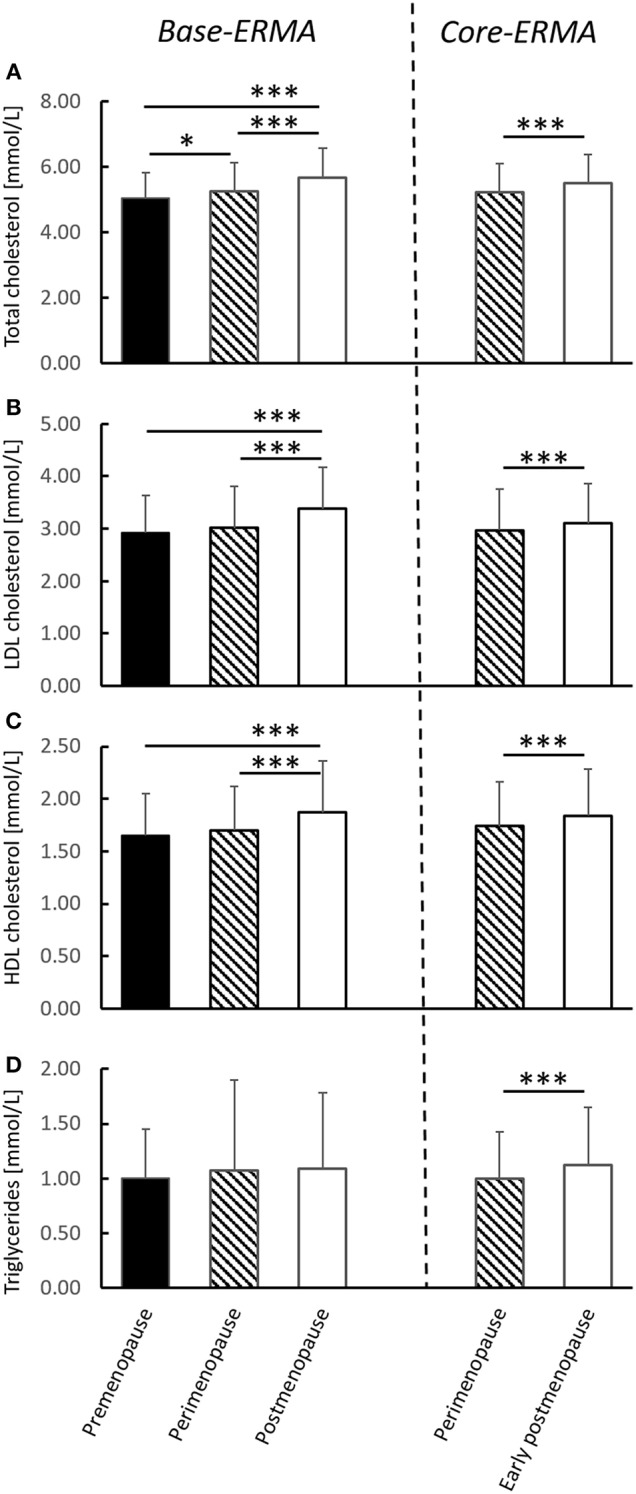
Serum CV risk factors from the cross-sectional (*base-ERMA*) and longitudinal (*core-ERMA*) study designs. Total cholesterol **(A)**, LDL-C **(B)**, HDL-C **(C)**, and triglyceride levels **(D)** from the pre-, peri- and post-menopausal groups in the *base-ERMA* design and from peri-menopause (baseline) and early post-menopause (follow-up) from the *core-ERMA* population. Recommended levels: Total cholesterol <5.2 mmol/L (<200 mg/dl), LDL <2.6 mmol/L (<100 mg/dl), HDL >1.5 mmol/L (60 mg/dl), and triglycerides <1.7 mmol/L (<150 mg/dl) (Mayo clinic, USA). Figures show average + SD, *p < 0.050, ***p < 0.001.

The serum CV risk factors are presented in Figure 2. In the *base-ERMA* population serum total cholesterol concentrations were significantly higher in the post-menopausal group than either of the other groups (*p* < 0.001). Total cholesterol levels were also higher in the peri-menopausal group compared to the pre-menopausal group (*p* < 0.050). Serum LDL-C and HDL-C concentrations followed similar trends, with higher concentrations in the post-menopausal group compared to the pre- and peri-menopausal subjects (*p* < 0.001). A similar trend was seen in the *core-ERMA* population, as serum total cholesterol, LDL-C, HDL-C, and triglyceride levels increased already at early post-menopause compared to the previous peri-menopausal values (*p* < 0.010). In the *core-ERMA* population thetriglyceride level also increased during the menopausal transition (*p* < 0.001).

Table 4 displays output from regression models of the *base-ERMA* population constructed with menopausal status as an independent predictor of serum CV risk factors. In addition, the *p*-values from the Generalized Estimating Equations (GEE) model comparing peri- and early post-menopausal levels of the studied parameters from the *core-ERMA* population are included in the table. The direction of the changes is the same in the *base*-ERMA and *core*-ERMA populations (β *coefficients for GEE models not shown*). In *base-ERMA*, menopausal status was positively associated with all three cholesterol measures in the univariate model, models that had been adjusted only for self-reported LTPA, and fully adjusted (adjusted for age, education, smoking, alcohol consumption, and body fat percentage) models (*p* < 0.001). The same analysis using accelerometer-measured LTPA as an adjustor is presented in the Supplementary Table 1. The longitudinal analysis using the core-ERMA population verified that the natural menopausal transition is the contributing factor. Also, in the adjusted models of *base-ERMA*, menopausal status was associated with triglyceride and fasting blood glucose levels (*p* < 0.001). In the *core-ERMA* population, the menopausal transition was positively associated with triglyceride levels (*p* < 0.001) but not with blood glucose levels. Leptin levels were measured only from the *base-ERMA* population and were not associated with menopausal status.

**Table 4:** Univariate and multivariate linear regression models with menopausal status as independent predictor of serum CV risk factors (*base-ERMA*).

	**Total Cholesterol [mmol/l]**					**LDL Cholesterol [mmol/l]**					**HDL Cholesterol [mmol/l]**				
	**β**	***p*-value**	***R*^**2**^**	***p*-value**	***p*-value**	**β**	***p*-value**	***R*^**2**^**	***p*-value**	***p*-value**	**β**	***p*-value**	***R*^**2**^**	***p*-value**	***p*-value**
	**Menopausal status**		**Full model**		GEE-model^*^	**Menopausal status**		**Full model**		GEE-model^*^	**Menopausal status**		**Full model**		GEE-model^*^
Univariate model	0.282	**<0.001**	0.080	**<0.001**	**<0.001**	0.242	**<0.001**	0.058	**<0.001**	**0.001**	0.200	**<0.001**	0.040	**<0.001**	**<0.001**
LTPA-adjusted model	0.282	**<0.001**	0.088	**<0.001**	**<0.001**	0.242	**<0.001**	0.077	**<0.001**	**0.001**	0.200	**<0.001**	0.065	**<0.001**	**<0.001**
Fully adjusted model^§^	0.238	**<0.001**	0.115	**<0.001**	**<0.001**	0.185	**<0.001**	0.118	**<0.001**	**0.001**	0.181	**<0.001**	0.148	**<0.001**	**<0.001**
	**Triglycerides [mmol/l]**					**Fasting blood glucose [mmol/l]**					**Leptin [ng/ml]**				
	**β**	***p*****-value**	***R***^**2**^	***p*****-value**	***p*****-value**	**β**	***p*****-value**	***R***^**2**^	***p*****-value**	***p*****-value**	**β**	***p*****-value**	***R***^**2**^	***p*****-value**	
	**Menopausal status**		**Full model**		GEE-model^*^	**Menopausal status**		**Full model**		GEE-model^*^	**Menopausal status**		**Full model**		
Univariate model	0.043	0.204	0.002	0.204	**<0.001**	−0.062	0.066	0.004	0.066	0.383	−0.018	0.588	0.000	0.588	
LTPA-adjusted model	0.042	0.202	0.032	**<0.001**	**<0.001**	−0.062	0.061	0.031	**<0.001**	0.383	−0.019	0.551	0.074	**<0.001**	
Fully adjusted model^§^	0.036	0.309	0.093	**<0.001**	**<0.001**	−0.076	**0.031**	0.076	**<0.001**	0.383	−0.035	0.177	0.519	**<0.001**	

* *GEE model of serum CV risk factors (core-ERMA). β, standardized regression coefficient; R^2^, Coefficient of determination, statistically significant coefficients are highlighted*.

§ *Model is adjusted for age, education level, smoking status, alcohol consumption, and percent body fat. Statistically significant findings (p < 0.05) are marked with bold in all of the tables*.

### *Base*- and *core-ERMA*: Association of LTPA With Serum CV Risk Factors

Table 5 displays output from regression models of the *base-ERMA* population and the *p*-values from the GEE model with self-reported LTPA (MET-h/day) as an independent predictor of serum CV risk factors. The direction of the changes is the same in the *base*-ERMA and *core*-ERMA populations (β *coefficients for GEE models not shown*). In *base-ERMA*, self-reported LTPA was positively associated with HDL-C and negatively associated with all of the other measured serum CV risk markers in univariate and fully adjusted models with or without percentage of body fat (adjusted for age, education, smoking, and alcohol consumption, *p* < 0.050) except for total cholesterol, which was negatively associated with self-reported LTPA in a fully adjusted model only when percent body fat was not included as an adjustor (*p* < 0.050). In *base-ERMA*, we were also able to investigate the associations between PA and serum CV risk factors by using accelerometer measured LTPA as a variable. The self-reported LTPA correlated positively both with accelerometer measured LTPA and total PA (β = 0.353, *p* < 0.001, and β = 0.270, *p* < 0.001; respectively). When regression models were built using accelerometer measured LTPA (total counts), which was available from 760 participants from *base-ERMA* population, the results obtained by using self-reported LTPA were verified for HDL-C, triglycerides, and leptin, but, possibly due to lower number of participants leading to reduced statistical power, not for other investigated CV risk factors. Accelerometer measured LTPA was positively associated with HDL-C and negatively associated with triglycerides and leptin levels (*p* < 0.05, Supplementary Tables 1, 2), and the same result was acquired when regression models were built using accelerometer measured total PA, which also included work time PA *(data not shown)*.

**Table 5:** Univariate and multivariate linear regression models with self-reported LTPA (MET-h/day) as independent predictor of serum CV risk factors (*base-ERMA*).

	**Total Cholesterol [mmol/l]**					**LDL Cholesterol [mmol/l]**					**HDL Cholesterol [mmol/l]**				
	**β**	***p*-value**	***R*^**2**^**	***p*-value**	***p*-value**	**β**	***p*-value**	***R*^**2**^**	***p*-value**	***p*-value**	**β**	***p*-value**	***R*^**2**^**	***p*-value**	***p*-value**
	**LTPA**		**Full model**		GEE-model^*^	**LTPA**		**Full model**		GEE-model^*^	**LTPA**		**Full model**		GEE-model^*^
Univariate model	−0.093	**0.005**	0.009	**0.005**	0.475	−0.137	**<0.001**	0.018	**<0.001**	**0.013**	0.157	**<0.001**	0.025	**<0.001**	**<0.001**
Fully adjusted model^£^	−0.048	0.157	0.115	**<0.001**	0.804	−0.072	**0.033**	0.118	**<0.001**	0.363	0.086	**0.009**	0.148	**<0.001**	**0.004**
Fully adjusted model without percent body fat^$^	−0.076	**0.019**	0.107	**<0.001**	0.622	−0.119	**<0.001**	0.095	**<0.001**	**0.038**	0.165	**<0.001**	0.084	**<0.001**	**<0.001**
	**Triglycerides [mmol/l]**					**Fasting blood glucose [mmol/l]**					**Leptin [ng/ml]**				
	**β**	***p*****-value**	***R***^**2**^	***p*****-value**	***p*****-value**	**β**	***p*****-value**	***R***^**2**^	***p*****-value**	***p*****-value**	**β**	***p*****-value**	***R***^**2**^	***p*****-value**	
	**LTPA**		**Full model**		GEE-model^*^	**LTPA**		**Full model**		GEE-model^*^	**LTPA**		**Full model**		
Univariate model	−0.173	**<0.001**	0.030	**<0.001**	**<0.001**	−0.166	**<0.001**	0.027	**<0.001**	**<0.001**	−0.272	**<0.001**	0.074	**<0.001**	
Fully adjusted model^£^	−0.098	**0.004**	0.093	**<0.001**	**0.002**	−0.098	**0.005**	0.076	**<0.001**	**0.001**	−0.061	**0.013**	0.519	**<0.001**	
Fully adjusted model without percent body fat^$^	−0.166	**<0.001**	0.045	**<0.001**	**<0.001**	−0.160	**<0.001**	0.037	**<0.001**	**<0.001**	−0.269	**<0.001**	0.075	**<0.001**	

* *GEE model of serum CV risk factors (core-ERMA). β, standardized regression coefficient; R^2^, Coefficient of determination, statistically significant coefficients are highlighted*.

£ *Model is adjusted for age, education level, smoking status, alcohol consumption, menopausal status, and percent body fat*.

$ *Model is adjusted for age, education level, smoking status, alcohol consumption and menopausal status. Statistically significant findings (p < 0.05) are marked with bold in all of the tables*.

In the longitudinal *core-ERMA* population, very similar results than in the cross-sectional *base-ERMA* population were obtained. Self-reported LTPA was positively associated with HDL-C, and negatively with triglyceride and fasting blood glucose levels in univariate and fully-adjusted models with and without percentage body fat (*p* < 0.050). Self-reported LTPA was also negatively associated with LDL-C levels in univariate and fully-adjusted models without body fat percentage in the *core-ERMA* population (*p* < 0.050), yet LTPA was not associated with total cholesterol levels. Leptin levels were measured only from the *base-ERMA* population.

## Discussion

The present study observed the menopause-associated changes in the serum lipid profile and the role of LTPA in decreasing the levels of these CV risk factors in healthy middle-aged women both with cross-sectional (*base-ERMA*) and longitudinal (*core-ERMA*) study designs. Our study showed that menopausal status was independently associated with higher serum total cholesterol, LDL-C and HDL-C concentrations, and an increasing trend was observed in each of these parameters with advancing menopause status while adjusting for age and LTPA both in *base-ERMA* and *core-ERMA* study designs. Our results highlight that the menopausal transition, not aging, drives the increase in total cholesterol and LDL-C levels, as well as an increase in the less-reported HDL-C level. In addition, we found that self-reported LTPA was associated with higher serum HDL-C and lower serum LDL-C levels as well as lower triglyceride and fasting blood glucose levels, independent of menopausal status in both of our study designs.

### Menopausal Status Is Associated With Serum CV Risk Factors Over Chronological Age

An accumulating body of literature shows that menopause is associated with unfavorable changes in lipid metabolism leading to an increased likelihood of developing metabolic syndrome and CVD (9–11, 23, 26). These changes can partially be opposed by estrogen-containing hormone therapy (HT). The mechanistic pathways of how estrogen and its receptors affect CVD have been studied extensively, yet mostly in pathophysiological conditions with animal models (27). In women, when started at post-menopause, orally administered estrogen reduces LDL-C levels (28, 29). The observed reduction in LDL-C seems to be a result of accelerated conversion of hepatic cholesterol to bile acids and increased expression of LDL receptors on cell surfaces leading to improved clearance of LDL from plasma (30, 31). Orally administered estrogen also increases HDL-C levels in post-menopausal women, which is due to decreased hepatic lipase activity and increased production of apolipoprotein A, the main protein component of HDL particles (29). However, there is a variety of HDL subclasses with different functions. Thus far, subparticle HDL_2_ is considered the most active in reverse cholesterol transport making it the key candidate in decreasing CV risk (32). Furthermore, in female mice, estrogen signaling through hepatocyte ERα was shown to regulate reverse cholesterol transport (33). The deletion of hepatocyte ERα both decreased the capacity of HDL to export cholesterol and increased serum total cholesterol level as well as HDL particle size. Yet we lack the information regarding which specific HDL particles increase with HT and only a few studies have investigated if menopause negatively affects functionality of HDL particles (34, 35).

Nevertheless, the existing literature has reported somewhat inconsistent associations of menopause with triglyceride and HDL-C levels. In contrast to our findings, two large European cross-sectional studies observed no significant differences in triglyceride or HDL-C levels after adjustment for age (14, 15), highlighting the role of chronological age on these particular serum CV risk factors. To further support this observation, a longitudinal American study found the increment in total cholesterol and LDL-C to follow the curve of FSH consistent with menopause-induced changes, while the increment in triglycerides and HDL-C were more gradual and followed the chronologic aging curve (23). In the present study, total cholesterol, LDL-C and HDL-C increased in both study designs and triglycerides in *core-ERMA* during the menopausal transition. The *Core-ERMA* study was designed to measure women as soon as possible after they became post-menopausal (verified by repeatedly high FSH levels), and thus was able to already capture changes in the measured biomarkers with this rather short (16 ± 8 months) follow up period only expanding to early post-menopause, strongly suggesting the underlying cause of changes in CV risk factors to be menopausal hormonal changes rather than aging. In previous studies, the age range of the subjects has been large (from 18 to 70-year-old women) and/or the determination of menopausal status has relied mostly on retrospective self-reports. In our present study, the age range of the subjects is very narrow (47–55 years) and menopausal status was defined both by using the bleeding diaries and blood hormonal level measurements, making our study design more robust in studying the effects of aging and menopausal status on CV risk factors. Our *core-ERMA* is the first longitudinal study to report that menopausal transition associates not only with higher LDL-C and total cholesterol levels, but also with higher triglycerides and HDL-C, independent of chronological aging, while controlling differences in physical activity.

Some other studies have found HDL-C levels to increase acutely before menopause and decrease after menopause (11, 23, 36). In our *base-ERMA* study, HDL-C was progressively higher across the stages of menopause from pre- to post-menopause as well as increased during the menopausal transition from peri-menopause to early post-menopause in *core-ERMA* (Figure 2). In line with our results, increased HDL-C levels with menopause have been previously reported (36–38), although this has been generally less highlighted compared to the menopausal increment in LDL-C. Traditionally, higher HDL-C levels have been considered to be atheroprotective. Therefore, it is not straightforward to understand why increased HDL-C accompanies the otherwise clearly negative menopausal changes in the lipid profile. There have been several attempts to explain this unexpected change, such as relatively high physical activity levels (38), yet all explanations point to the fact that low HDL-C levels do not seem to be the main factor causing increased CVD risk or metabolic syndrome in post-menopausal women (37). In our study, the results of the regression and GEE models did not change after adjusting for LTPA, i.e., the changes in lipid profile were not due to decrease in LTPA level. Woodard et al. (36) speculated that the higher HDL-C at post-menopause may be explained by functional and compositional changes in HDL particles (36, 39). More specifically, among women, the profile of the HDL and LDL particle sizes seems to shift around menopause or over the menopausal transition toward more atherogenic lipoprotein subtypes (13, 26, 36). Indeed, it seems that the atheroprotective effect of HDL may be weaker in women after menopause (36, 40). Therefore, in the future, measuring lipoprotein particles and separately analyzing lipoprotein subclasses with simultaneous analysis of blood hormone levels may provide additional insight into the association between lipids and CVD risk factors at menopause.

### LTPA May Attenuate Menopause-Associated Unfavorable Changes in Serum CV Risk Factors

More recently, studies have concentrated on how PA affects the detrimental changes associated with menopause. The current literature has rather controversial results regarding the effect of exercise on blood lipid profiles of menopausal women. Some controlled intervention trials show an improvement on blood lipid levels after either resistance or aerobic exercise training in post-menopausal women (41–44). Yet in all these intervention studies, exercise was combined with either estrogen-containing hormone replacement therapy (HT) or weight-reducing diets, or the study subjects were dyslipidemic at the start of the intervention. Two more recent studies (20, 45) with healthy subjects showed a positive effect of exercise on the blood lipid profile. Behall et al. (45) compared the type of exercise to effects on plasma lipids in overweight, pre- and post-menopausal women, and found that the type of exercise (resistance or aerobic) was more important to post-menopausal than pre-menopausal women, aerobic exercise being more efficient on lowering serum total cholesterol level (45). Mandrup et al. (20) found that 3 month high-intensity training improved several CVD factors in healthy, non-obese post-menopausal women, including lower diastolic blood pressure, total cholesterol, and LDL-C (20). Nonetheless, some intervention studies have shown no improvement in blood lipid levels after exercise training (46–49), even with training interventions similar to those associated with positive effects in other studies. In all, exercise appears to result in improvements in the blood lipid profile of overweight or dyslipidemic post-menopausal subjects, yet results are less consistent with subjects that are healthy at baseline.

In the present study designs, self-reported LTPA level was associated with higher HDL-C and lower LDL-C levels in fully adjusted models that did not include percent body fat (Table 5). In the *base-ERMA* population, LTPA was no longer associated with total cholesterol when percent body fat was included in the models, although it still was associated with lower LDL-C and higher HDL-C. Excess body weight has been associated with higher serum total cholesterol, LDL-C and triglyceride levels, indicating that high body weight, or high body fat percentage, is linked to dyslipidemia (50–52). This association most likely causes the difference in the predictive power of LTPA with and without body fat percentage in cholesterol levels in our study setup. Correlation analysis showed that increasing PA levels were associated with decreased total and LDL-C concentrations and increased HDL-C. Self-reported LTPA was also a highly significant predictor of triglycerides, fasting blood glucose, and leptin (Table 5). Moreover, we found no interaction between LTPA and menopausal status in the *base-ERMA* population serum CV risk factor regression models, suggesting that LTPA associates similarly among middle-aged women regardless of their menopausal status. This is important comprehensive evidence on several different serum CV risk factors indicating that PA beneficially affects the CVD risk of healthy middle-aged women. In particular, PA has the potential to combat the progressive increment in LDL-C levels with advancing menopause status, which, in combination with its association with other biomarkers of CVD risk, may further alleviate disease risk.

Even though high LTPA evidently has health benefits, our results revealed that a similar LTPA level than at pre-menopause was not sufficient to prevent the unfavorable lipid profile changes associated with the menopausal transition. This observation is in line with previous literature, where exercise intervention was either accompanied with HT and/or diet (42, 43), or exercise was done at high-intensity level (20) to gain healthier blood lipid profiles in post-menopausal women. Hence, to obtain a clinically relevant effect on serum CV risk factors, a greater LTPA dose and/or simultaneous changes in diet may be necessary in healthy, post-menopausal women.

### Study Strengths and Limitations

This study has several important strengths to note. It is part of a large, comprehensive cohort study designed to characterize the changes that occur in women at the time of menopause using previously validated research methods. It relied on a combination of bleeding diaries and serum FSH levels, rather than on self-report alone to accurately classify women into the proper menopausal groups. Participants were subjected to extensive questionnaires, physical measurements, and lab testing to effectively identify the variables exerting an effect on the subjects' CVD risk. In addition, we were able to obtain data from longitudinal study design from the same participants as in the cross-sectional study design, further strengthening our findings. The discontinuation rate was low, and the participant number was larger than in many studies that have previously attempted to address related issues.

The current study also has some limitations. Although our study provides valuable information about changing CV risk profiles associated with different menopausal status and degree of PA, its clinical relevance is somewhat limited by the lack of prospective, patient-centered outcomes, such as CV event incidence, CV event mortality, and all-cause mortality.

We collected retrospective survey data on the occurrence of myocardial infarction and stroke. This historical data was helpful to assess the baseline cardiovascular health of the participants. However, if accelerated atherosclerosis is indeed the primary mechanism by which menopause increases CVD risk, it is unlikely that the adverse event incidence would change quickly enough to be captured in this study. Currently we are performing a follow-up study from the ERMA study population, termed EsmiRs (www.jyu.fi/esmirs/en). EsmiRs will provide 4-year follow-up since the baseline of the ERMA study allowing us to investigate if the serum lipid profile continues to worsen in this population. However, even longer follow-ups are needed to define how detrimental the currently revealed early signs of worsening lipid profiles due to menopause will clinically turn out to be.

This study was designed to intensively characterize the differences seen due to menopause among women aged 47–55 years and during the menopausal transition from peri-menopause to early post-menopause and identify differences and early changes in CV risk factors that may help to explain the well-documented increases in CVD in post-menopausal women later in their lives. In the future, more sophisticated metabolomics analyses may further increase the understanding of the relation of LTPA and menopausal transition to CV risk factors (53).

In the present study, we chose to use self-reported LTPA in the association analyses to retain higher sample sizes and to utilize an estimation of LTPA activity representing a longer period than captured by the usual 7-day use of the accelerometer. Using accelerometer-measured LTPA decreases the sample size in cross-sectional study design by 126 subjects, which greatly decreases the power of the analysis, as shown in Supplementary Tables 1, 2. The self-reported questionnaire data focused on capturing the average physical activity level over an extended period of time, which is what will influence the cardiovascular risk profile. The accelerometers, by contrast, offered only a 7-day snapshot of the physical activity. However, it is known that self-reports are prone to over-estimation of PA. Therefore, we also used accelerometers to measure LTPA and did not find significant differences between group means of total activity counts. Self-reported LTPA correlated significantly both with accelerometer measured LTPA and total PA. Also, the group difference we observed in accelerometer measured vigorous LTPA (highest level in post-menopausal women) would not favor the worse cholesterol profile observed in the post-menopausal group. Therefore, it is unlikely that a possible reporting bias in self-reported LTPA would lead to overestimation of associations observed between menopausal status or LTPA and serum CV risk factors.

Finally, the population studied here was highly homogenous, as every woman enrolled identified herself as Caucasian/white. Therefore, it is unknown the extent to which these results are generalizable to women of other ethnic groups or those living in developing countries with less robust health systems.

## Conclusions

In conclusion, this large-scale cohort study of middle-aged women complemented by longitudinal investigations of women over menopause found that the menopausal transition is associated with increases in serum levels of total cholesterol, LDL-C, and HDL-C that are independent of age, percent body fat and LTPA. The level of LTPA, in turn, is an independent predictor of the variation in LDL-C and HDL-C concentrations between the participants and higher PA level is associated with cardioprotective effects such as lower LDL-C and higher HDL-C, as well as lower levels of triglycerides, fasting blood glucose, and leptin. These results suggest that the menopausal transition is a stronger determinant of serum CV risk factors than chronological age. We observed that LTPA may attenuate menopause-associated atherogenic changes of healthy middle-aged women, yet it does not seem to entirely compensate for the alterations in the serum lipid profile. Hence, to obtain a clinically relevant effect on serum CV risk factors, a greater LTPA dose and/or or simultaneous dietary intervention may be necessary in healthy, post-menopausal women.

## Materials and Methods

### Study Population

The ERMA study is a population-based cohort study comprising of Caucasian women aged 47–55 years living in the city of Jyväskylä (Finland) and neighboring municipalities (Figure 1). The collection of the cross-sectional *base-ERMA* data proceeded in three phases (pre-questionnaire, group assignments and health screen questionnaire, and laboratory visit with psychological and physiological measures), as detailed in Kovanen et al. (25). Of the total cohort, 82% was approached by a postal inquiry and invited to take part in the study and to return the pre-questionnaire enabling screening for exclusion. Of the initial 1,393 participants who completed the group assignments and health screen questionnaire, 389 were pre-menopausal, 474 peri-menopausal, and 530 post-menopausal. The response rate for the postal inquiry was 45%. Those willing to participate and fulfilling the inclusion criteria were asked to fill out a menstrual diary for at least 12 weeks before the first laboratory visit for initial health screening and blood sampling. Participants who currently used, had used during the past 3 months estrogen-containing contraceptives or other estrogen-containing medications, used lipid lowering medication, had had bilateral oophorectomy, were pregnant or lactating, had polycystic ovary syndrome or other conditions affecting ovarian function, had a BMI > 35 kg/m^2^ (based on self-reported height and weight), or had any musculoskeletal disorder seriously affecting everyday PA were excluded. Additionally, participants having conditions or use of medications affecting daily mental or physical function or systemic hormone or inflammatory status were excluded (25). Between the initial health screening questionnaire and the following physiological and psychological assessments, 471 participants were excluded and 36 discontinued the study. Finally, participants without available data for all variables used in the regression models, due to missing values or study discontinuation (4 participants), as well as those taking prescription lipid-lowering medications (23 participants) were excluded from the final analysis of the current study. After all exclusions and dropouts, 886 women remained in the cross-sectional *base-ERMA* study setup: 230 pre-menopausal, 368 peri-menopausal, and 288 post-menopausal. The *base-ERMA* population was recruited to explore the associations of menopausal status and LTPA on serum CV risk factors in a population based, large-scale study design.

Of the 368 peri-menopausal women in the *base-ERMA* study, 193 went through the natural menopausal transition during the 16 ± 8 month follow-up period, forming the longitudinal *core-ERMA* study design (Figure 1). The *core-ERMA* design was reconstructed from *base-ERMA* to enable the examination of a smaller cohort of the exact same individuals from peri-menopause to early post-menopause, minimizing confounding factors. The same exclusion criteria as for the *base-ERMA* study were applied for the *core-ERMA* study, with the exception that participants who had begun using hormone-replacement therapy (*n* = 12) were not excluded from the analysis.

### Ethics Statement

The ERMA study was approved by the Ethics Committee of the Central Finland Health Care District (KSSHP Dnro 8U/2014). All study participants gave written informed consent. The study protocol followed good clinical and scientific practice and the Declaration of Helsinki.

### Blood Sampling and Serum CV Risk Factor Profiling

Fasting (12 h) blood samples were taken from the antecubital vein in supine position between 7:00 and 10:00 a.m. and during the first 5 days of the menstrual cycle, when the cycle was predictable. For serum separation, whole blood was left to clot for 30 min at room temperature and centrifuged at 2,200 × g before aliquoting and storing the sera at −80°C. Serum FSH and estradiol levels were determined using IMMULITE® 2000 XPi (Siemens Healthcare Diagnostics, UK) according to manufacturer's instructions. Blood glucose, total cholesterol, LDL-C, HDL-C and triglycerides were measured using KONELAB 20 XTi analyzer (Thermo Fischer Scientific, Finland) according to manufacturer's instructions. Leptin was measured using Human Leptin ELISA-kit (RD191001100, BioVendor, Czech Republic) according to manufacturer's instructions.

### Determination of Menopausal Status

The determination of menopausal status as pre-menopausal, peri-menopausal, or post-menopausal is based on the menstrual diary and measured FSH level as has been presented in detail earlier (25). The main procedure follows the Stages of Reproductive Aging Workshop (STRAW)+10 guidelines, which define different menopausal states as follows; pre-menopausal women have regular menstrual cycle with variable FSH and estradiol, peri-menopausal women have irregular menstrual cycle with elevated FSH and lowered estradiol and post-menopausal women have at least 12 months of amenorrhea with high (> 30 IU/L) FSH and low estradiol (54). However, due to study feasibility reasons, participants were asked to keep bleeding diaries for a minimum of 3 months before blood was drawn for hormone assessment, which may have led some peri-menopausal women to be erroneously categorized as post-menopausal. By the time of the second laboratory visit, women categorized as naturally post-menopausal had provided diaries showing an average of 150 ± 71 days without menstrual bleeding and FSH levels 83 ± 30 IU/L.

Another potential source for misclassification is the unreliability of the bleeding pattern. Therefore, for participants whose menstrual bleeding pattern was not completely natural, i.e., they had undergone hysterectomy or were using progesterone-containing contraceptives, the group assignment relied on the FSH assessment, but with more stringent cut-off values (given in parentheses) than those used for participants with natural menstrual bleeding. A participant was categorized as pre-menopausal, if she had a regular bleeding pattern and FSH <17 IU/L (15 IU/L), as peri-menopausal, if she had irregular or no bleeding and FSH 17–30 IU/L (15–39 IU/L) and as post-menopausal, if she had no menstrual bleeding and FSH > 30 IU/L (>39 IU/L). A few women who had very high FSH levels (>130 IU/L), but had still occasional bleeding days, were also categorized as post-menopausal.

The *core-ERMA* participants, who at baseline were determined to be peri-menopausal, revisited our laboratory in 3–6 months intervals and their hormone levels were measured. Before determining women had reached early post-menopausal status, the FSH levels needed to be high in two consecutive measurements.

### Socio-Demographic and Life-Style Factors and Health-Related Variables

Education, smoking, and use of alcohol were assessed by standard questionnaires. Education was classified as primary, secondary or tertiary based on the highest education level reported by the participant. Participants were categorized based on their smoking habits as never, quitter, or current smoker. According to reported use of alcohol, participants' mean consumption per week was calculated. Information regarding diseases, use of medication, and gynecological issues was self-reported using standard, pre-structured questionnaires. The presence of chronic conditions and use of prescribed medication were confirmed via nurse's interview according to a pre-structured questionnaire and current prescriptions. The Anatomical Therapeutic Chemical (ATC) Classification System by the World Health Organization (WHO) was used for drug classification, with A10: drugs used in diabetes, C07: β*-*blocker*s*, C08: calcium channel blockers, and C09: agents acting on the renin–angiotensin (RAAS) system (https://www.whocc.no/atc_ddd_index). Only those drug classes known to influence the serum CV risk factors used in cardiovascular risk assessments are reported here.

### Anthropometrics, Body Composition, Physical Fitness, and Physical Activity

Anthropometrics and body composition were measured between 7:00 and 10:00 a.m. after overnight fasting and physical fitness after light breakfast in the same day as reported in Kovanen et al. (25). Briefly, body weight was measured with a beam scale and height by a stadiometer with the participant wearing only undergarments and BMI was calculated as weight (kg)/height squared (m^2^). Body fat percentage and lean body mass were assessed with a multifrequency bioelectrical impedance analyzer (InBody^TM^ 720; Biospace, Seoul, Korea). Physical fitness was assessed by 6-min walking test (55). The test was performed on a 20-m indoor track, and participants were instructed to complete as many laps as possible within 6 min to assess submaximal exercise tolerance and aerobic capacity.

For PA assessments, both self-reports and accelerometer measures were used. The PA questionnaire is described previously (56). Briefly, the metabolic equivalent (MET) of PA was calculated as a product of intensity, duration and frequency from 3 questions of LTPA. The following MET values were used: 4 (for PA intensity corresponding to walking), 6 (vigorous walking to jogging), 10 (jogging), and 13 (running). The total LTPA was expressed as MET-h/day. In addition PA was measured with 7-day use of GT3X+ or wGT3X+ ActiGraph accelerometer (Pensacola, Florida, USA) as reported previously (57). Participants were instructed to wear the monitors on their right hip for 7 consecutive days during their waking hours except while bathing or doing other water-based activities. They were also provided with a diary and instructed to record their wake-up time, working hours, and periods, when the monitor was removed for longer than 30 min. Raw acceleration data were collected at 60 Hz, filtered and converted into 60-s epoch counts. A customized Excel-based program was used for further data analysis. Mean time spent at different PA intensities i.e., light, moderate, and vigorous was calculated for each participant using tri-axial vector magnitude cut-points of 450, 2,690, and 6,166 cpm, respectively (57, 58). The cut-point of upper vector magnitude limit was 25,000 cpm. LTPA was separated from whole day PA based on the notes of working hours in the activity diaries. The PA data were then normalized to 10-h wake time for LTPA and 16-h wake time for total PA (59). For association analyses, the self-reported LTPA was used. This enabled using the whole dataset (*n* = 886) with higher statistical power, as accelerometer measured PA was available only from 760 subjects. Furthermore, the self-reported questionnaire data estimated the average physical activity level over an extended period of time instead of the accelerometer measured, 7-day measurements of the PA.

### Statistics

Data are presented as mean and standard deviation (SD) for continuous variables and as frequency (n) and percentage (%) for categorical variables. The normality of the variables was assessed using the Shapiro-Wilks test. As only a few variables met the normal distribution criteria, differences between the menopausal groups were investigated using the Kruskal-Wallis and Mann-Whitney tests. To estimate the independent association of predictor variables with the serum lipid variables in the *base-ERMA* design, univariate, and multivariate linear regression models were constructed. Before building up the multivariate regression models, homoscedasticity was tested, the autocorrelation was tested with Durbin-Watson test, and multicollinearity was inspected with variance inflation factor to fit into the recommended range. Correlations between self-reported and accelerometer-measured LTPA and total PA were investigated using Spearman's correlation. The change in characteristics of peri-menopausal women, who went through the menopausal transition during follow-up time (the *core-ERMA* design), were examined using Wilcoxon test. To estimate the independent association of predictor variables with the serum CV risk factors in the *core-ERMA* design, the Generalized Estimating Equations (GEE) model was used. In the GEE model, the baseline measurement was used for age, education, smoking status, alcohol consumption, fat percentage and self-reported LTPA. Data analysis was carried out using IBM SPSS Statistics software version 24 (Chicago, IL, US), and the level of significance was set at *p* < 0.05.

#### Sensitivity Analyses

Separate sensitivity analyses were performed for the *base-ERMA* population, with the participants using progesterone-based contraceptives in the past 3 months or having had hysterectomies excluded from the analysis in order to evaluate if unreliability issues in the menstrual bleeding patterns or the complete lack of bleeding had influenced our primary analyses. The results of the sensitivity analyses did not markedly differ from our primary analysis (*data not shown*). Separate sensitivity analyses were also performed for the *core-ERMA* population excluding the participants using HT (*n* = 12), and results did not significantly differ from our primary analysis (*data not shown*).

## Data Availability

The datasets used and/or analyzed during the current study are available from the corresponding author on reasonable request.

## Author Contributions

MJ and SK wrote the manuscript and did the statistical analysis of the studied parameters. MH helped drafting the manuscript and ran the regression analysis. TT is responsible for the accelerometer data. PA for the gynecological data. UK for the medical examinations. SS, VK, and EL planned the ERMA study and provided financial support. EL and UK supervised drafting the manuscript and interpretation of the data regarding the manuscript. All authors read and approved the final manuscript.

### Conflict of Interest Statement

The authors declare that the research was conducted in the absence of any commercial or financial relationships that could be construed as a potential conflict of interest.
