# Supplementary material for: Menopausal Status and Physical Activity Are Independently Associated With Cardiovascular Risk Factors of Healthy Middle-Aged Women: Cross-Sectional and Longitudinal Evidence
Source: Front Endocrinol (Lausanne). 2019 Aug 30;10:589. doi: 10.3389/fendo.2019.00589 (PMC6729112; doi:10.3389/fendo.2019.00589)
Supplement: Supplementary file 1 [file Table_1.docx]

# Supplementary information

## *Base-ERMA:* Regression models built with accelerometer measured LTPA

**Table 1:** Univariate and multivariate linear regression models with menopausal status as independent predictor of serum CV risk factors (*base-ERMA*). Participants not having accelerometer measured PA data (n = 126) excluded.

|  | **Total Cholesterol [mmol/l]** | | | | **LDL Cholesterol [mmol/l]** | | | | **HDL Cholesterol [mmol/l]** | | | |
| --- | --- | --- | --- | --- | --- | --- | --- | --- | --- | --- | --- | --- |
|  | **β** | **p-value** | **R^2^** | **p-value** | **β** | **p-value** | **R^2^** | **p-value** | **β** | **p-value** | **R^2^** | **p-value** |
|  | **Menopausal status** | | **Full model** | | **Menopausal status** | | **Full model** | | **Menopausal status** | | **Full model** | |
| Univariate model | 0.271 | **< 0.001** | 0.073 |  | 0.239 | **< 0.001** | 0.057 |  | 0.197 | **< 0.001** | 0.039 |  |
| LTPA (accelerometer)-adjusted model | 0.270 | **< 0.001** | 0.074 | **< 0.001** | 0.242 | **< 0.001** | 0.060 | **< 0.001** | 0.188 | **< 0.001** | 0.080 | **< 0.001** |
| Fully adjusted model^§^ | 0.231 | **< 0.001** | 0.103 | **< 0.001** | 0.198 | **< 0.001** | 0.096 | **< 0.001** | 0.176 | **< 0.001** | 0.150 | **< 0.001** |
|  |  |  |  |  |  |  |  |  |  |  |  |  |
|  | **Triglycerides [mmol/l]** | | | | **Fasting blood glucose [mmol/l]** | | | | **Leptin [ng/ml]** | | | |
|  | **β** | **p-value** | **R^2^** | **p-value** | **β** | **p-value** | **R^2^** | **p-value** | **β** | **p-value** | **R^2^** | **p-value** |
|  | **Menopausal status** | | **Full model** | | **Menopausal status** | | **Full model** | | **Menopausal status** | | **Full model** | |
| Univariate model | 0.029 | 0.432 | 0.001 |  | -0.084 | 0.020 | 0.007 |  | -0.025 | 0.484 | **< 0.001** |  |
| LTPA (accelerometer)-adjusted model | 0.035 | 0.329 | 0.022 | **< 0.001** | -0.084 | 0.021 | 0.007 | 0.066 | -0.015 | 0.674 | 0.054 | **< 0.001** |
| Fully adjusted model^§^ | 0.019 | 0.608 | 0.092 | **< 0.001** | -0.109 | **0.004** | 0.076 | **< 0.001** | -0.041 | 0.130 | 0.535 | **< 0.001** |
|  |  |  |  |  |  |  |  |  |  |  |  |  |

β = standardized regression coefficient, R^2^ = Coefficient of determination, statistically significant coefficients are highlighted

^§^ Model is adjusted for age, education level, smoking status, alcohol consumption and percent body fat
