# Supplementary material for: Menopausal Status and Physical Activity Are Independently Associated With Cardiovascular Risk Factors of Healthy Middle-Aged Women: Cross-Sectional and Longitudinal Evidence
Source: Front Endocrinol (Lausanne). 2019 Aug 30;10:589. doi: 10.3389/fendo.2019.00589 (PMC6729112; doi:10.3389/fendo.2019.00589)
Supplement: Supplementary file 2 [file Table_2.docx]

# Supplementary information

## *Base-ERMA:* Regression models built with accelerometer measured LTPA

## Table 2: Univariate and multivariate linear regression models with accelerometer measured LTPA (total counts) as independent predictor of CV risk factors (*base-ERMA*). Participants not having accelerometer measured PA data (n = 126) excluded

|  | **Total Cholesterol [mmol/l]** | | | | **LDL Cholesterol [mmol/l]** | | | | **HDL Cholesterol [mmol/l]** | | | |
| --- | --- | --- | --- | --- | --- | --- | --- | --- | --- | --- | --- | --- |
|  | **β** | **p-value** | **R^2^** | **p-value** | **β** | **p-value** | **R^2^** | **p-value** | **β** | **p-value** | **R^2^** | **p-value** |
|  | **LTPA (accelerometer)** | | **Full model** | | **LTPA (accelerometer)** | | **Full model** | | **LTPA (accelerometer)** | | **Full model** | |
| Univariate model | 0.028 | 0.447 | 0.001 |  | -0.041 | 0.264 | 0.002 |  | 0.212 | **< 0.001** | 0.045 |  |
| Fully adjusted model^£^ | 0.046 | 0.203 | 0.103 | **< 0.001** | -0.007 | 0.846 | 0.096 | **< 0.001** | 0.142 | **< 0.001** | 0.150 | **< 0.001** |
| Fully adjusted model without percent body fat^$^ | 0.020 | 0.561 | 0.093 | **< 0.001** | -0.045 | 0.198 | 0.073 | **< 0.001** | 0.202 | **< 0.001** | 0.093 | **< 0.001** |
|  | **Triglycerides [mmol/l]** | | | | **Fasting blood glucose [mmol/l]** | | | | **Leptin [ng/ml]** | | | |
|  | **β** | **p-value** | **R^2^** | **p-value** | **β** | **p-value** | **R^2^** | **p-value** | **β** | **p-value** | **R^2^** | **p-value** |
|  | **LTPA (accelerometer)** | | **Full model** | | **LTPA (accelerometer)** | | **Full model** | | **LTPA (accelerometer)** | | **Full model** | |
| Univariate model | -0.142 | <0.001 | 0.020 |  | -0.011 | 0.769 | <0.001 |  | -0.232 | **< 0.001** | 0.054 |  |
| Fully adjusted model^£^ | -0.076 | **0.036** | 0.092 | **< 0.001** | 0.057 | 0.120 | 0.076 | **< 0.001** | -0.054 | **0.038** | 0.535 | **< 0.001** |
| Fully adjusted model without percent body fat^$^ | -0.139 | **< 0.001** | 0.030 | **0.001** | -0.004 | 0.922 | 0.019 | **0.022** | -0.229 | **< 0.001** | 0.056 | **< 0.001** |

β = standardized regression coefficient, R2 = Coefficient of determination, statistically significant coefficients are highlighted

£ Model is adjusted for age, education level, smoking status, alcohol consumption, menopausal status and percent body fat

$ Model is adjusted for age, education level, smoking status, alcohol consumption and menopausal status
